# High *Aedes* spp. larval indices in Kinshasa, Democratic Republic of Congo

**DOI:** 10.1186/s13071-021-04588-7

**Published:** 2021-02-01

**Authors:** Francis Wat’senga Tezzo, Sylvie Fasine, Emile Manzambi Zola, Maria del Carmen Marquetti, Guillaume Binene Mbuka, Gillon Ilombe, Richard Mundeke Takasongo, Nathalie Smitz, Juan Andre Bisset, Wim Van Bortel, Veerle Vanlerberghe

**Affiliations:** 1 Unit of Entomology, Department of Parasitology, National Institute of Biomedical Research, 5345 Avenue De la Démocratie, Gombe, Kinshasa, Democratic Republic of the Congo; 2 Department of Vector Control, Instituto Medicina Tropical Pedro Kourí (IPK), Avenida Novia del Mediodía, KM 6 1/2, La Lisa, Havana, Cuba; 3 Department of Biology, Royal Museum for Central Africa (BopCo), Leuvensesteenweg 13-17, Tervuren, Belgium; 4 Outbreak Research Team, Institute of Tropical Medicine (ITM), Nationalestraat 155, Antwerp, Belgium; 5 Unit of Entomology, Biomedical Science Department, Institute of Tropical Medicine (ITM), Nationalestraat 155, Antwerp, Belgium; 6 Tropical Infectious Disease Group, Public Health Department, Institute of Tropical Medicine (ITM), Nationalestraat 155, Antwerp, Belgium

**Keywords:** Kinshasa, Central Africa, *Aedes*, Survey, Chikungunya, Democratic Republic of Congo

## Abstract

**Background:**

Dengue, yellow fever, chikungunya and Zika are among the most important emerging infectious vector-borne diseases worldwide. In the Democratic Republic of Congo (DRC), increases in cases of dengue and outbreaks of yellow fever and chikungunya have been reported since 2010. The main vectors of these arboviruses, *Aedes aegypti* and *Aedes albopictus*, have been reported in DRC, but there is a lack of detailed information on their presence and spread to guide disease control efforts.

**Methods:**

In 2018, two cross-sectional surveys were conducted in Kinshasa province (DRC), one in the rainy (January/February) and one in the dry season (July). Four hundred houses were visited in each of the four selected communes (N’Djili, Mont Ngafula, Lingwala and Kalamu). Within the peri-domestic area of each household, searches were conducted for larval habitats, which were then surveyed for the presence of *Aedes* larvae and pupae. A subset of the immature specimens were reared to adults for morphological identification followed by DNA barcoding of the specimens to validate identifications.

**Results:**

The most rural commune (Mont Ngafula) had the highest pupal index (number of *Aedes* spp. pupae per 100 inspected houses) at 246 (20) pupae/100 houses, and Breteau index (BI; number of containers positive for immature stages of *Aedes* spp. per 100 households) at 82.2 (19.5) positive containers/100 houses for the rainy (and dry) season, respectively. The BI was 21.5 (4.7), 36.7 (9.8) and 41.7 (7.5) in Kalamu, Lingwala and N’Djili in the rainy (and dry) season, respectively. The house index (number of houses positive for at least one container with immature stages of *Aedes* spp*.* per 100 inspected houses) was, on average, across all communes, 27.5% (7.6%); and the container index (number of containers positive for immature stages of *Aedes *spp*.* per 100 inspected containers) was 15.0% (10.0%) for the rainy (and dry) season, respectively. The vast majority of *Aedes*-positive containers were found outside the houses [adjusted odds ratio 27.4 (95% confidence interval 14.9–50.1)]. During the dry season, the most productive containers were the ones used for water storage, whereas in the rainy season rubbish and tires constituted key habitats. Both *Ae. aegypti* and *Ae. albopictus* were found. *Anopheles* larvae were found in different types of *Aedes* larval habitats, especially during the rainy season.

**Conclusions:**

In both surveys and in all communes, the larval indices (BI) were higher than the arbovirus transmission threshold values established by the World Health Organization. Management strategies for controlling *Aedes* in Kinshasa need to target the key types of containers for *Aedes* larvae, which are mainly located in outdoor spaces, for larval habitat destruction or reduction.
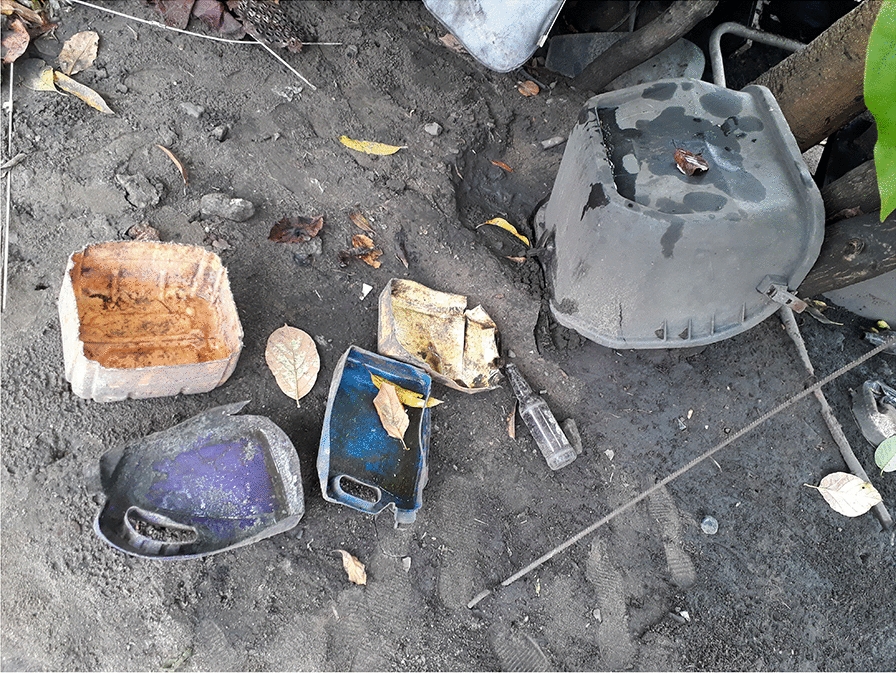

## Background

Arboviruses cause a variety of diseases, such as dengue, yellow fever, chikungunya and Zika, which are among the most important emerging infectious diseases worldwide [1–3]. The distribution of these diseases and their transmitting vectors have been well characterized for Latin America and Southeast Asia [4–6], but our understanding of arbovirus ecology in sub-Saharan Africa remains limited [7–9]. Dengue seroprevalence studies have shown that there is, or has been, dengue virus circulation in Cameroon, demonstrated by 12.5% immunoglobulin G (IgG) positivity; in Burkina Faso, demonstrated by 36% IgG positivity; in Nigeria, demonstrated by 45% IgG positivity [7]; and in Tanzania, demonstrated by 50.6% IgG positivity [10]. However, few reports have shown the importance of dengue as a cause of acute fever in these settings. This is also a case for the presence of dengue in the Democratic Republic of Congo (DRC), where the virus was found in stored samples: a dengue antigen test was positive for three suspected chikungunya cases in Kinshasa in 2012 [11]; 0.6% of dried blood spots taken during a Demographic Health Survey were positive in 2013–2014 [12]; and 3.5% of suspected cases of yellow fever in the Bas Congo region between 2002 and 2013 were dengue positive [13]. More recently, in 2015–2016, in Mont Ngafula (a suburban area of Kinshasa), 8.1% of acute fever cases were dengue positive [14], and 30.2% of the 342 study participants had dengue IgG antibodies. Although no outbreak of dengue has thus far been reported in DRC, in the neighboring country of Angola there was an outbreak of dengue with an estimated attack rate of 10% in 2013 [15]. By contrast, there have been apparent outbreaks of chikungunya, such as the one in Kenya in 2004 [16], in Tanzania in 2013 [17], in Mozambique in 2018 [18], in Brazzaville (DRC) in 2011 [19] and 2019 [20], and in Kinshasa, capital of DRC in 2000 [21], 2012 [22] and 2019 [23]. Such outbreaks can affect large populations, i.e. 67% of the population in Kenya [24]. Besides dengue and chikungunya, other alpha-, flavi- and bunyaviruses were also found in mosquito samples (*Aedes* and *Culex*) in Kinshasa in 2014 [25]. Zika has been rarely detected in sub-Saharan Africa [26], but several yellow fever outbreaks, with the last major one in 2016, have been described [27].

Information on the presence and distribution of *Aedes* mosquitoes in sub-Saharan Africa is even more difficult to find than information on the pathogens discussed above. This lack of entomological data forces recourse to suitability maps, which are based on mathematical models, to estimate arbovirus transmission risk [28]. However, measures of real *Aedes* spp*.* infestation levels would give more reliable insights for both risk and mitigation strategies [29]. Both *Aedes aegypti* and *Aedes albopictus* are found in sub-Saharan Africa; *Ae. aegypti* is native to the region, whereas *Ae. albopictus* was introduced from Southeast Asia [30] in early 2000 [31], and specifically into Kinshasa (DRC) in 2018 [32]. Both species have been detected in domestic environments [30], such as in Kinshasa, a megacity with a high population density and movement of people, but precise larval indices for *Aedes* remain unknown. Without knowing the main locations of *Aedes* larval habitats or the types of containers in which *Aedes* become adults, it is impossible to define effective larval source management strategies for Kinshasa.

In this study, we evaluate larval indices of *Aedes* spp*.* together with the characteristics of their preferred larval habitats, to help produce evidence-based guidance for *Aedes* control efforts and to provide insight into the potential for the local mosquito population to transmit arboviruses in Kinshasa, the capital of DRC.

## Methods

### Settings

**Fig. 1:**
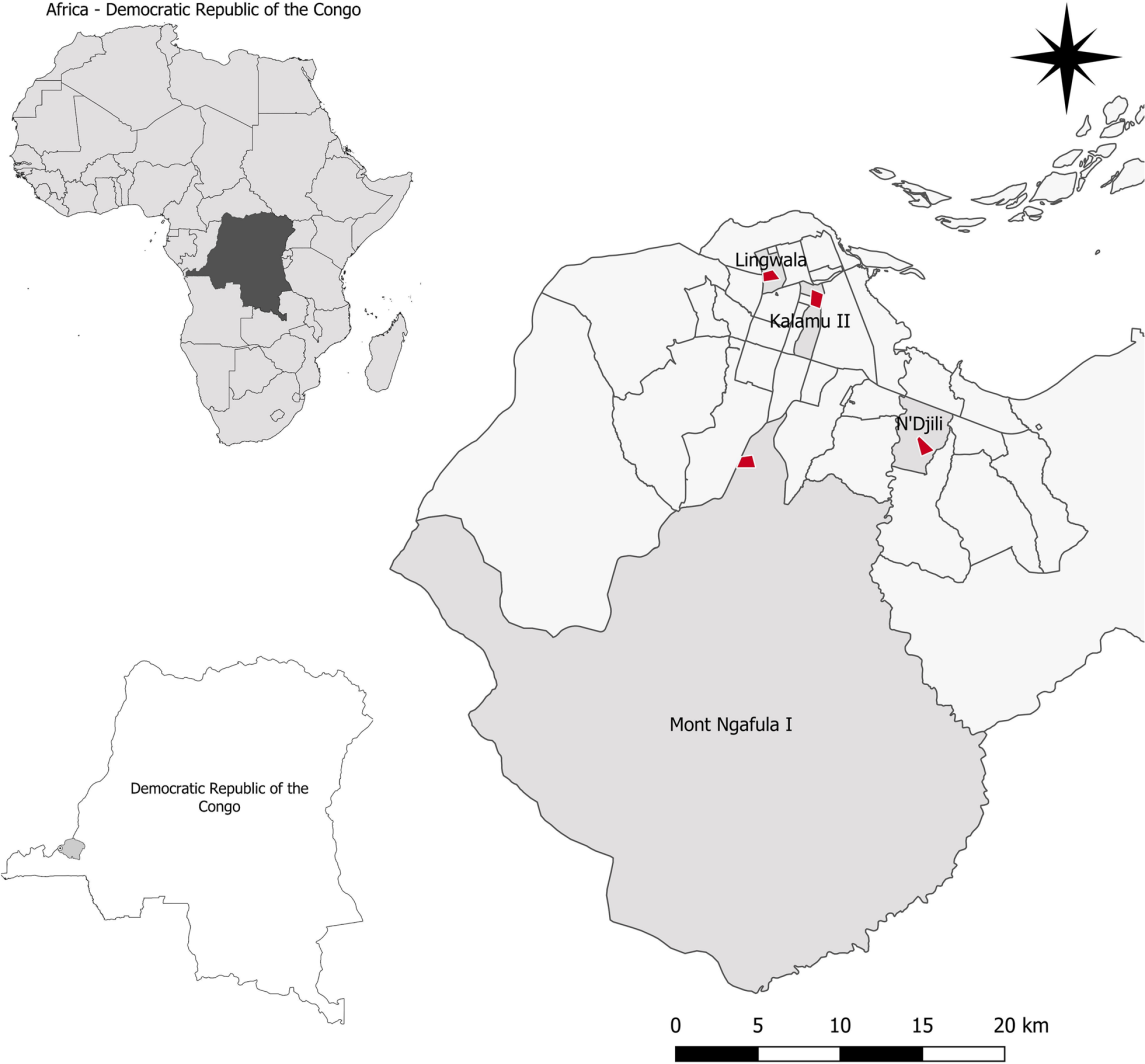
Maps indicating the survey sites. The four survey communes (*light grey*) with sampling areas indicated by* red dots*, in Kinshasa, 2018

The surveys took place in Kinshasa, the capital city of DRC, which is located in Central Africa. Kinshasa lies at 279 m above sea level and is characterized by a tropical climate with a rainy season between October and May, and a dry season from June to September. The average temperature varies between 18 and 32 °C, and the average monthly rainfall varies between 2 and 222 mm, in the dry and rainy seasons, respectively. Kinshasa covers an area of 9965 km^2^ and has an estimated population of almost 12 million people. The city is administratively subdivided into 24 communes, which are grouped into four districts: Tshangu in the east, Lukunga in the north, Mont Amba in the southeast and Funa in the center west. In this study, four communes were purposively selected to capture diverse ecological, urbanization, and epidemiological conditions (i.e. history of arbovirus outbreaks) and water supply systems (Fig. 1).

N’Djili is a peri-urban commune in the east of the city, within Tshangu district, where many informal economic activities, specifically vehicle repair shops, are located. Urban infrastructure and services, such as waste water infrastructure and garbage collection, are deficient. Almost all (97%) of the houses have a water supply system in their compound, but quality, volume and availability of water are problematic for a high number of them. The population density of this area is estimated at 39,000 people/km^2^.

Kalamu II is a commune in the center of town, within Funa district, and is mainly residential. The main economic activity is technical service provision. It has an estimated population density of 47,000 people/km^2^.

Mont Ngafula I is situated in the south of the city, bordering Mont Amba district, and is a typical semi-urban area with an estimated population density of 730 people/km^2^. It is geographically characterized by hills (and erosion) and small valleys. The main economic activity is agriculture and the selling of agricultural products in Kinshasa city. Mont Ngafula I is emblematic of unplanned urbanization, as it has a deficient water supply system in terms of both the rate of supply (i.e. as low as two times per week) and waste water disposal.

Lingwala is a commune in the center of the city, within Lukunga district, and has a large number of informal street markets. It is a more urbanized area than the others, and has a fairly good water supply. The population density is estimated at 33,000 people/km^2^.

### Study design and data collection

Two cross-sectional surveys were undertaken, one in the rainy season (18 January–16 February 2018) and one in the dry season (2–27 July 2018). To identify 10% of the houses that were positive for *Aedes* spp*.* mosquitoes at 80% power, 3% precision and allowing for a 5% α-error, 400 houses needed to be surveyed in each survey site. In each of the four selected communes, one neighborhood was randomly chosen (all the neighborhoods per commune were listed, followed by a random number selection procedure) as the study site. Each day, 80 houses were inspected following a systematic sampling approach: random points were identified on a landmark (a roundabout or a main road) by each team as their starting point from which to enter (smaller) streets. With a sampling interval of three houses, starting on the right side of a street, each of the four teams inspected the selected houses up to a maximum of 20 houses/day. If the quota of 20 houses had not been met when the street came to an end, the team turned back and inspected the houses on the other side of the street in the same manner until the sample quota had been reached. Each selected house was inspected inside and outside. If there was more than one house per compound, a random house was chosen for inspection, but the entire outside area of the compound was inspected. The next day, the next street (going left from the street sampled on the previous day) was sampled. By following this procedure, representative sampling was achieved. When the inspection of one commune had been completed, the four entomological teams went to another commune and followed the same methodology. The inspection of all the communes was achieved within a period of 4 weeks. Each entomological survey team consisted of three people: one person previously trained by the entomology department of the Institut National de Recherche Biomédicale (INRB), one entomologist from the INRB (the supervisor), and one community health worker.

In each compound, all water-holding containers were inspected and if immature stages of mosquitoes (i.e. larvae or pupae) were observed, they were collected in plastic bottles (one bottle per larval habitat) and transported to the laboratory at INRB for identification to genus (*Anopheles*, *Aedes*, *Culex*). The location, category and positivity/negativity for mosquitoes of each container were recorded. For larvae, only positivity and negativity was recorded; for pupae, the number of pupae was counted per positive habitat. The surveys were implemented identically, but as samples were randomly selected, the houses had equal probability of inclusion in one, both, or neither survey. Both surveys were largely realized by the same field team members.

### Morphological and DNA-based species identification

Each day, a random sample of 50 *Aedes* larvae/pupae were reared to adults in an insectarium to allow species identification using morphological keys [33, 34]. F0 adults were stored at – 20 °C for DNA barcoding to validate the morphological identification of *Ae. aegypti* and *Ae. albopictus* and confirm the presence of the identified species in Kinshasa. Five specimens of each species were randomly selected per survey site for DNA barcoding. DNA barcoding is a technique based on the amplification of a standard barcode—the partial mitochondrial cytochrome *c* oxidase subunit I (COI) gene for animals. Sanger sequencing of the 658-base pair COI standard barcode was performed using LCO1490 and HCO2198 universal primers [35, 36]. Amplifications were carried out in a 20-µl reaction mixture containing 2 µl of DNA template, 2 µl of 10× buffer, 1.5 mM MgCl_2_, 0.2 mM dNTP, 0.4 µM of each primer, and 0.03 units/µl of Platinum Taq DNA Polymerase (Invitrogen). PCR products and negative controls were checked on a 1.5% agarose gel, using a ultraviolet transilluminator and the MidoriGreen Direct (NIPPON Genetics Europe) method. Positive amplicons were purified using the ExoSAP-IT protocol and sequenced in both directions on an ABI 3230xl capillary DNA sequencer using BigDye Terminator v3.1 chemistry (ThermoFisher Scientific). The generated sequences were then compared to a library of reference sequences. Specimens were identified by analyzing their percentage sequence similarity with the reference sequences under the assumption that genetic diversity is lower within than between species. A rooted neighbor-joining tree was constructed including a sub-selection of the *Ae. albopictus* and *Ae. aegypti* barcodes available from online repositories, together with the newly generated haplotypes (full details of the protocol can be found in Additional file 2).

### Data analysis

Data were entered into the Microsoft Access database and 5% of the data were manually validated to detect errors. Data cleaning was undertaken and the types of recipients regrouped into categories, adapted from guidelines used in dengue-endemic regions [37], as follows: water storage tanks or cisterns (> 15 l); small water containers used for daily kitchen and cleaning activities (< 15 l); rubbish and discards; natural tree and bamboo holes; artificial containers that are used by households and cannot be destroyed (e.g. animal drinking pots); used tires; natural ground surface pools. Data were analyzed using IBM SPSS statistics, version 25. We calculated, per round of visits and per commune, the house index (HI; number of houses positive for at least one container with immature stages of *Aedes* spp. per 100 inspected houses), Breteau index (BI; number of containers positive for immature stages of *Aedes* spp*.* per 100 inspected houses), container index (CI; number of containers positive for immature stages of *Aedes *spp*.* per 100 inspected containers), and pupal index (number of *Aedes* spp. pupae per 100 inspected houses). The relative contribution to pupal productivity, defined as the total number of pupae of *Aedes* spp*.* per category of larval habitat divided by the total number of pupae of *Aedes* spp. collected per commune and per survey round, was calculated. A descriptive analysis was done. In order to evaluate the factors determining *Aedes* spp*.* immature stage positivity, a logistic regression model was used and associated variables were identified based on a backwards conditional model, taking into account clustering at the household level by inserting the household identification variable as a random factor in the model.

The number of larval habitats with at least one immature stage of *Anopheles *spp*.* was enumerated and its proportional importance calculated for each season and respective commune.

## Results

In the surveys, a total of 1678 and 1598 houses were sampled in the rainy and dry season, respectively. In the rainy season, 5079 water-holding containers (potential larval habitats) were inspected compared with 1657 in the dry season. The average number of containers per household varied across communes (*p* < 0.001), e.g. in the rainy season, there was an average of 1.4 (SD 1.3) in Kalamu, 2.0 (SD 1.7) in Lingwala, 2.9 (SD 2.3) in Mont Ngafula and 5.3 (SD 2.6) in N’Djili. In the rainy and dry season, 65.9% and 78.3% of the containers, respectively, were observed outside the sampled houses, i.e. in the open space within the compound (*p* < 0.001). The distribution of the types of containers per location, commune and season is given in more detail in Additional file 1 (Table 1).

**Table 1:** Entomological indices for *Aedes *spp*.* for the four survey sites in the rainy and dry seasons, Kinshasa 2018

		Total	Lingwala	Ndjili	Mont Ngafula	Kalamu
No. of containers inspected	Rainy/dry season	5079/1657	821/180	2550/665	1164/634	544/178
Container index (%)^a^	Rainy season	14.98	17.90	7.84	28.18	15.81
	Dry season	10.02	21.67	4.51	12.30	10.67
No. of houses inspected	Rainy/dry season	1678/1598	400/399	479/399	399/400	400/400
Breteau index (no. positive containers/100 houses)^b^	Rainy season	45.35	36.75	41.75	82.21	21.50
	Dry season	10.39	9.77	7.52	19.50	4.75
House index (%)^c^	Rainy season	27.53	22.25	27.97	44.86	15.00
	Dry season	7.63	7.02	6.52	13.25	3.75
Pupal index (no. pupae/100 houses)^d^	Rainy season	128.00	90.00	126.00	246.00	50.00
	Dry season	15.00	13.00	9.00	20.00	19.00

^a^Number of containers positive for immature stages of *Aedes *spp*.* per 100 inspected containers

^b^Number of containers positive for immature stages of *Aedes* spp*.* per 100 inspected houses

^c^Number of houses positive for at least one container with immature stages of *Aedes* spp. per 100 inspected houses

^d^Number of *Aedes* spp. pupae per 100 inspected houses

**Fig. 2a–g:**
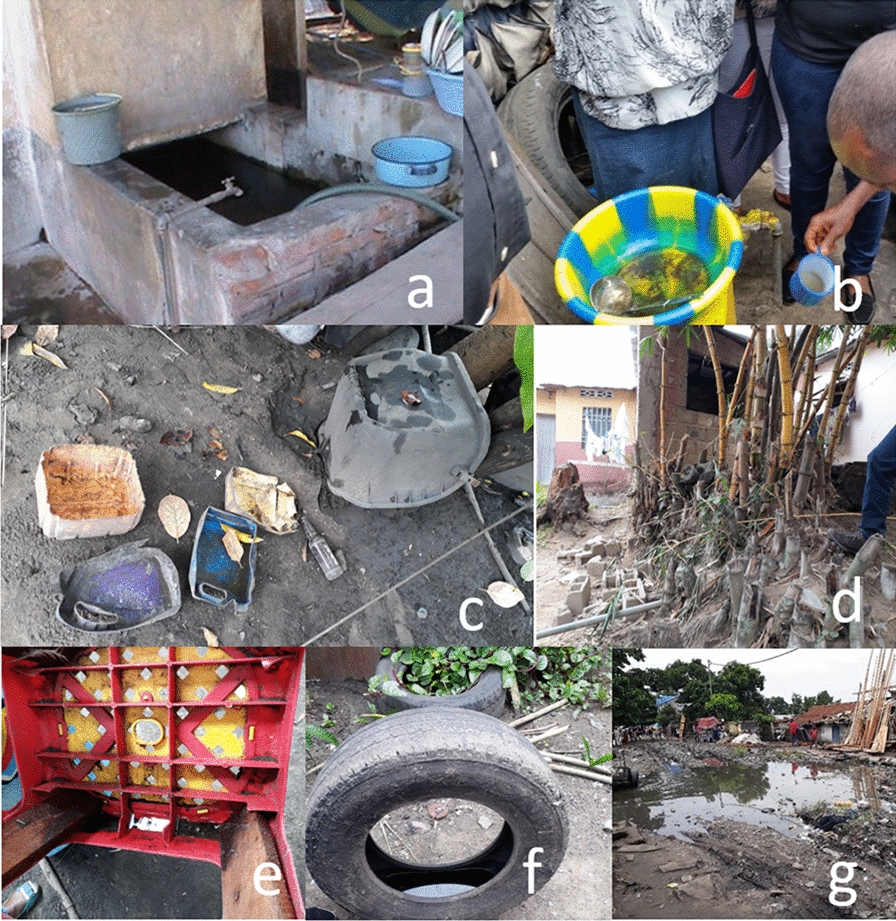
Photos of various types of larval habitat identified/investigated in the study area. **a** Water storage tanks or cisterns (> 15 l); **b** small water containers (< 15 l) used for daily kitchen and cleaning activities; **c** rubbish and discards; **d** natural tree and bamboo holes; **e** artificial containers that are used by households and cannot be destroyed (e.g. animal drinking pots); **f** used tires; **g** natural ground surface pools

*Aedes* larval indices were higher in the rainy than in the dry season (*p* < 0.001; Table 2), with a BI of 45.35 versus 10.39 positive containers/100 houses, a CI of 14.9% versus 10.02% and a HI of 27.53% versus 7.63%, respectively (Table 1). Mont Ngafula, a rural sub-urban area on the southern edge of Kinshasa had the highest infestation levels amongst all the visited communes, with a BI of 82.21 and 19.50 positive containers/100 houses in the rainy and dry season, respectively, about four times higher than those of Kalamu, a commune that lies within the heart of the city. In the rainy season, the pupal index reached 246 pupae/100 houses in Mont Ngafula, 126 in N’Djili, 90 in Lingwala, and 50 in Kalamu (Table 1). In the rainy season, 99.3% of the positive larval habitats were outdoors versus 96.4% in the dry season. A wide variety of containers were occupied by *Aedes* mosquitoes as aquatic habitat: water storage tanks, small water deposits, rubbish/dicards, bamboo/tree holes, non-destroyable artificial containers, used tires, natural ground pools (Fig. 2). Tires were treated as a separate group, as they were frequently present and it was difficult to know if they had been put aside for re-use/temporary storage or for destruction.

**Table 2:** Determinants of *Aedes* spp*.-*positive larval habitats in Kinshasa, 2018

Parameter	Category	Total	Positive [*n* (%)]	Multivariate	
				Odds ratio (95% confidence interval)	*p*-value
Season	Rainy	5079	761 (15.0)	1.98 (1.63–2.40)	< 0.001
	Dry	1657	166 (10.0)	1	
Commune	Kalamu	722	105 (14.5)	0.97 (0.74–1.28)	0.857
	Lingwala	1001	186 (18.6)	1.53 (1.20–1.96)	0.001
	Mont Ngafula	1798	406 (22.6)	2.67 (2.19–3.25)	< 0.001
	N’Djili	3215	230 (7.2)	1	
Position	Exterior	4646	916 (19.7)	27.36 (14.9–50.1)	< 0.001
	Interior	2090	11 (0.5)	1	
Container type	Water storage tanks	1080	94 (8.7)	1	
	Small water containers (small water deposits)	4373	395 (9.0)	0.99 (0.77–1.27)	0.918
	Rubbish	533	134 (25.1)	1.89 (1.39–2.55)	< 0.001
	Bamboo hole	5	0 (0)	0	1
	Artificials not destroyable	18	4 (22.2)	0.998 (0.32–3.13)	0.997
	Used tires	710	296 (41.7)	4.60 (3.50–6.06)	< 0.001
	Ground pools	17	4 (23.5)	2.06 (0.62–6.79)	0.236

**Fig. 3:**
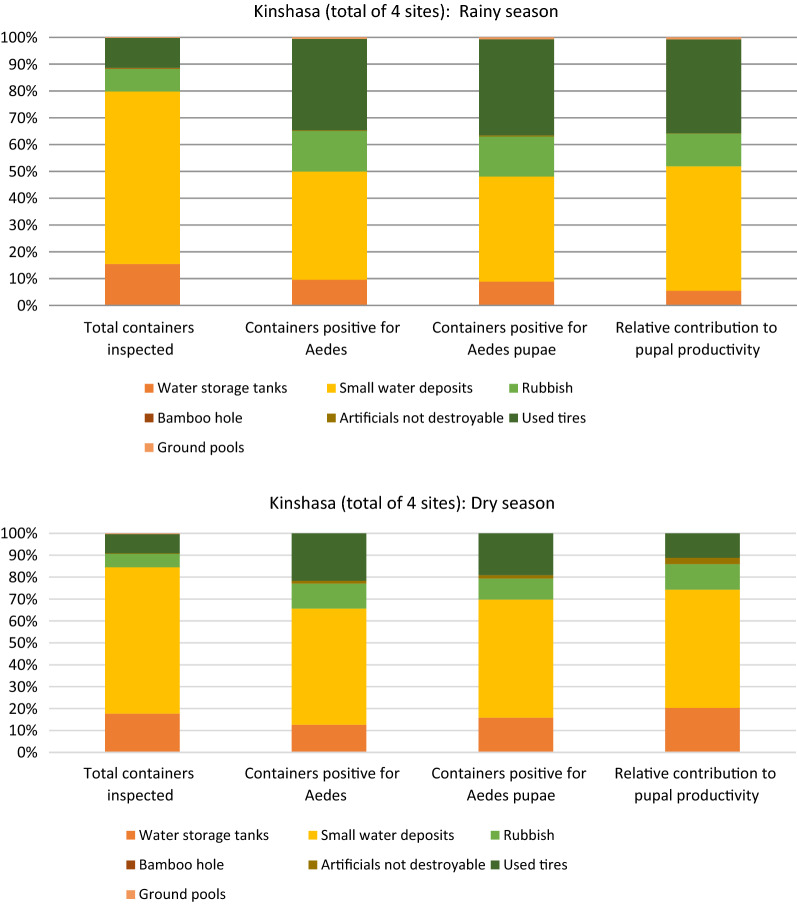
Productivity of the habitats for immature stages of *Aedes* spp*.*, Kinshasa, 2018

**Fig. 4:**
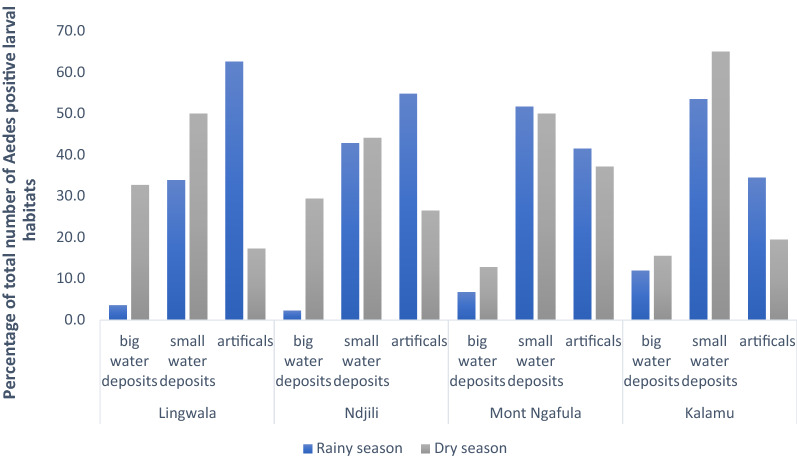
Geographical and seasonal differences of the most productive habitats for *Aedes* spp. larvae, Kinshasa, 2018

We observed a statistically significant difference between the pupal productivity of larval habitats in the rainy and dry seasons [adjusted odds ratio (aOR) 3.73, 95% confidence interval (2.21–6.31); *p* < 0.001]. In the dry season, 20.3% of pupal production was in water storage tanks versus 5.5% in the rainy season, which indicated seasonal variability in aquatic habitat preference of the vectors (Fig. 3). In the rainy season, 64.3% of all inspected containers were small water containers, but these were only responsible for 46.4% of the pupal production, whereas used tires, representing only 11.1% of the inspected containers, were responsible for 35.0% of the pupal production. The containers used for water storage (big and small) contributed relatively more to pupal productivity in the dry season than in the rainy season. Furthermore, the pupal productivity of artificial containers (mainly rubbish) was different across communities (*p* < 0.001) and season (*p* < 0.001) (Fig. 4).

Positivity for *Aedes* was higher in the rainy than in the dry season with an aOR of 1.98 (95% confidence interval 1.6–2.4), and was about 27 times [aOR 27.4 (95% confidence interval 14.9–50.1)] higher outdoors than indoors (*p* < 0.001). Mont Ngafula and Lingwala were statistically significantly more infested than N’Djili (*p* < 0.001; Table 2). The types of water container most associated with *Aedes* infestation were used tires [aOR 4.6 (95% confidence interval 3.5–6.1)] and rubbish/discards [aOR 1.9 (95% confidence interval 1.4–2.5)], rather than water storage tanks (Table 2).

**Fig. 5:**
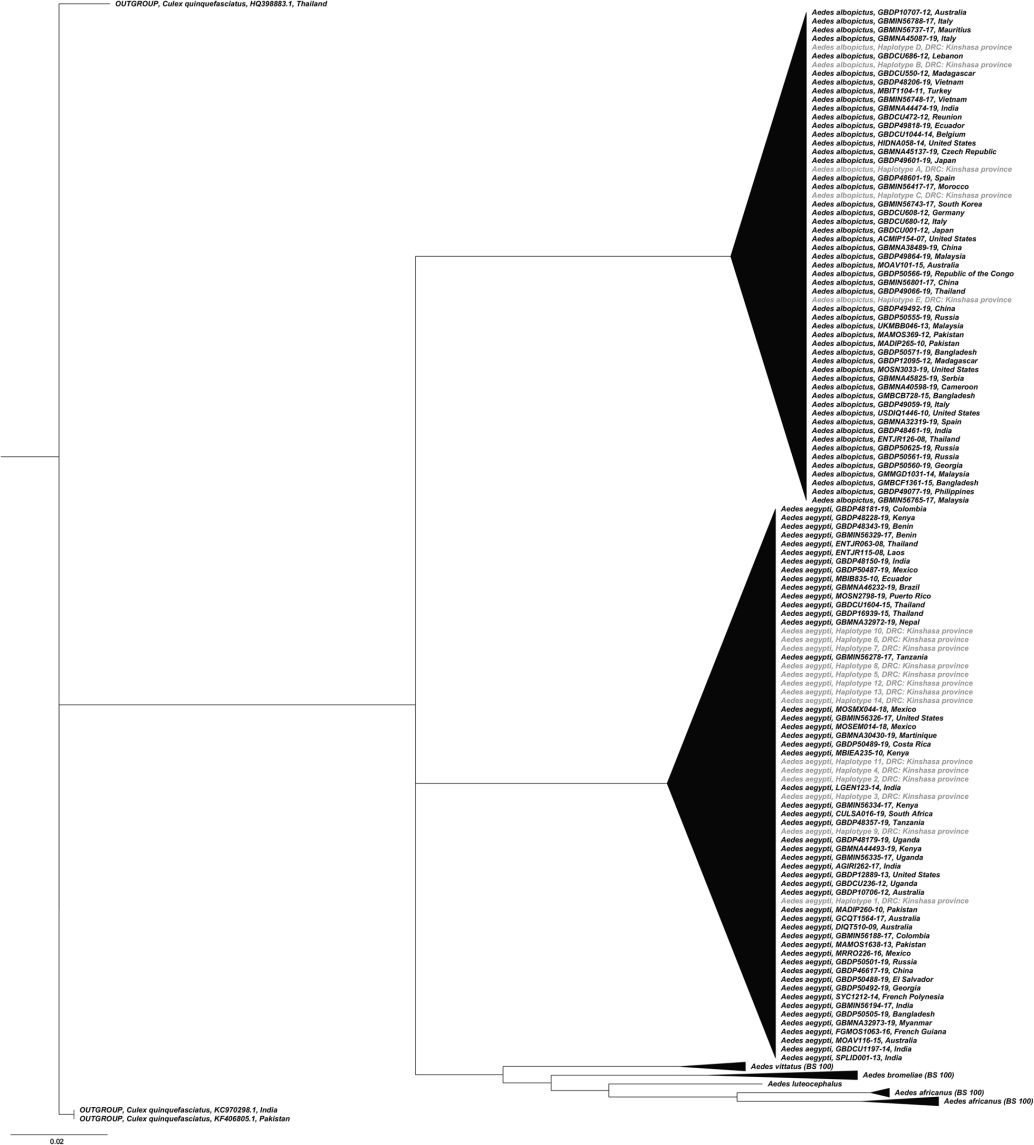
Neighbor-joining tree including the six medically important *Aedes* species of the subgenus *Stegomyia* occurring in the Afrotropical region. The generated haplotypes of *Aedes albopictus* and *Aedes aegypti* specimens of the Democratic Republic of Congo are highlighted in* grey*

Based on morphological identification, F0 adult *Ae. aegypti* and *Ae. albopictus* were present in both seasons and at all study sites. Morphological identification was validated by comparing the generated sequences of a subset of specimens against the BOLD Identification System with Species Level Barcode Records. The obtained similarity percentages ranged from 99.69 to 100%. The five and 14 haplotypes of *Ae. albopictus* and *Ae. aegypti*, respectively, only clustered with sequences from conspecific specimens collected worldwide (Fig. 5); this was supported by maximum bootstrap results (Additional file 2: Figure S1). The generated sequences were deposited in GenBank with the following accession numbers: MT345349-MT345426.

**Fig. 6:**
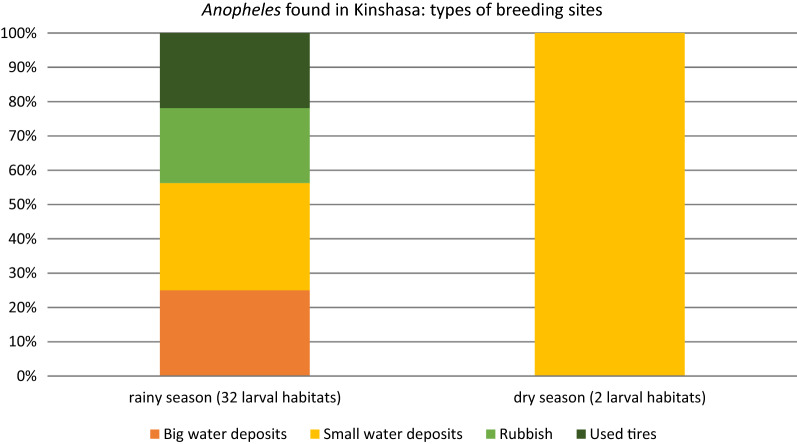
Types of habitats where *Anopheles* spp*.* larvae were encountered in the Kinshasa survey sites, 2018 (*n* = 32 mixed larval habitats positive for *Anopheles*)

**Fig. 7:**
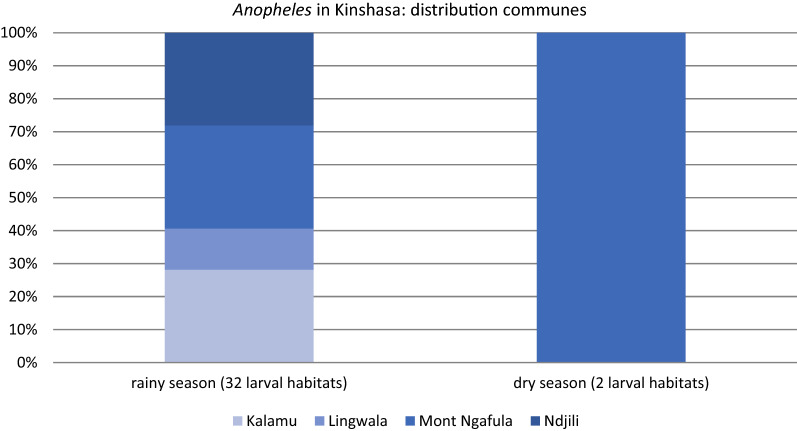
Distribution of habitats positive for *Anopheles* spp. larvae in the four survey communes in the rainy and dry season, Kinshasa, 2018 (*n* = 32 mixed larval habitats positive for *Anopheles*)

Among containers positive for *Aedes* spp*.* immature stages, 9.46% and 9.06% also contained immature stages of other genera, such as *Culex* and *Anopheles*, in the rainy and dry season, respectively. In 99.3% of the outdoor recipients, and specifically in water storage tanks in the rainy season and in trash in the dry season, habitats were shared. In the rainy season, a total of 32 *Aedes* larval habitats were positive for *Anopheles* versus only two in the dry season. *Anopheles* were found in big and small water deposits, rubbish and used tires (Fig. 6). In the rainy season, *Anopheles* were observed in all communes, whereas in the dry season, *Anopheles* larvae were only found in small water deposits in Mont Ngafula, the most rural commune of the four survey sites (Fig. 7).

## Discussion

In both surveys, in all the communes studied, the larval indices (HI, CI, and BI) were higher than the arbovirus transmission threshold values established by the World Health Organization (BI of 5) [38, 39]. As the BI was, on average, 45 positive containers/100 houses in the rainy season, and the HI, on average, 27%, it is clear that one household can have different *Aedes* spp.-positive larval habitats. Should an arbovirus be introduced into Kinshasa in the future, the high larval and pupal *Aedes* densities found in the present study suggest that transmission could rapidly occur and lead to a major outbreak of disease, such as that seen for chikungunya in 2019 [23].

The standardized procedure used for the surveys in the four different communes of Kinshasa during the rainy and dry seasons is the major strength of this study. The members of the entomological team were trained before the study, and were largely the same in both surveys. A weakness of this study is that the surveys took place over only 1 year and only once per season. As the inspection of the larval habitats depended on the rigor and professionalism of the team doing the fieldwork, quality control was established by ensuring that a supervisor was available in the field site during the survey, and regular additional quality control was also undertaken by international members of the survey team. Due to operational issues, we were not in a position to identify all the larvae to species level; this was only done for a small subsample of larvae, hence we could not calculate the specific relative importance of *Ae. aegypti* and *Ae. albopictus*, but only observe a tendency for their equal presence. Neither could we calculate which species has a predilection for which container type. *Ae. aegypti* and *Ae. albopictus* display different vector competence for different arboviruses, and more detailed information on the occurrence of each vector would allow us to develop more precise control measures in the case of a specific arbovirus outbreak. *Aedes aegypti*, which originated in Africa, is the main vector of arboviruses globally, but its vector competence is highly variable and depends on the vector population, the virus isolate and the ecological context [28]. The presence of *Ae. albopictus*, which is an exotic species in Africa, might change the epidemiology of a number of arboviruses in Africa [40]. In several chikungunya epidemics, *Ae. albopictus* has been shown to be the main driver of transmission of the chikungunya virus, especially in the case of the East/Central/South African lineage with A226V mutation, as shown in a recent outbreak in Kinshasa [41].

In a place like Kinshasa, where dengue is rarely reported [11–14], and there are only sporadic outbreaks of chikungunya and yellow fever [21, 23, 42], the high *Aedes* larval and pupal indices found in the present study are unexpected. The indices observed here are comparable to those reported for other African settings, e.g. southeastern Tanzania has a HI of 4.9–6.6, and CI of 14.6–18.9 [43]; Burkina Faso has a HI of 70, a CI of 35 and a BI of 10 [44]; northwest Ethiopia has a HI of 25.5, a CI of 32.9 and a BI of 48.4 [45]; Mozambique has a CI of 22 [46]; and Angola has a HI of 4.3–27.9, a CI of 2.1–9.3 and a BI of 5.8–42.2 [47]. However, the *Aedes* larval and pupal indices found in the present study are much lower than those observed in Kenya during a dengue outbreak in 2013–2014, where the BI reached a value of 270 positive containers/100 houses [48].

In contrast to findings from Latin-America [49], in Kinshasa, immature stages of *Aedes* were found in 19.7% (916/4646 containers) of the outdoor containers versus 0.5% (11/2090) of the indoor containers. These differences between the number of immature stages of *Aedes* indoors and outdoors are characteristic of findings for other African countries [50]. The prevalence of *Aedes* larval habitats outdoors together with the behavior of *Aedes* in this context (remaining outside in the backyard or in the open in front of a house and blood feeding on humans during the day) suggests that there is a close relationship between humans and these mosquitoes, which favors the life cycle of *Aedes* spp. [51]. The low presence of immature stages of *Aedes* inside the houses could also have been a consequence of the rapid use of water from, and the cleaning of, the containers found there. These results indicate that management strategies for the control of *Aedes* in Kinshasa need to target outdoor spaces for the destruction or reduction of larval habitats.

Used car tires, water storage tanks and other artificial larval habitats (type rubbish/discards) were the main types of container chosen by *Aedes* mosquitoes for oviposition, which is in agreement with other studies conducted on these mosquitoes in Africa [44–48, 50, 52]. Water storage tanks were found to be the most productive containers for the pupal stage of *Aedes*, which is the non-feeding stage preceding the adult stage [53]. The water storage tanks are always kept partially or fully filled with water irrespective of rainfall due to the deficient water supply system, which makes them a preferred larval habitat, especially in the dry season, even though they are constantly subject to anthropogenic activity. In the rainy season, larval habitats were favored by rainfall in all the survey sites, and the containers typically filled with rainwater were the most productive ones for *Aedes* pupae. Old tires are illustrative of this, as while they only represent 11% of the potential larval habitats, in the rainy season, about 35% of all of the pupae were found in them. The temperature, humidity and reduced light inside tires create a suitable environment for *Aedes* reproduction, and when tires are stored or have been discarded for a long period of time without being scrubbed, larvae can proliferate in them [54–56]. Under such conditions, eggs can also remain attached to tires for a long period of time, and tires thus play a role in the preservation of the *Aedes* mosquito population throughout the dry season [57]. The disproportionate importance of certain containers for pupae—and hence for the production of adult *Aedes*—identified in the present study is useful as it indicates which key larval habitats should be targeted in management strategies that are designed to decrease arbovirus transmission risk.

In this study, *Aedes* spp., which are vectors of chikungunya, dengue, Zika and yellow fever, were dominant in the potential larval habitats that were inspected in and around the houses. Other mosquito genera were also found, such as *Culex* and *Anopheles*. While *Culex* may share larval habitats with urban species of *Aedes*, it is unusual to find *Anopheles* species together with *Aedes* [58]. *Anopheles* usually prefer other types of larval habitats, such as ponds with static fresh water, and are not particularly attracted to small containers [59]. The presence of *Anopheles* in urban settings was previously thought to be associated with urban agriculture, as previously seen in Mont Ngafula [60]. However, we found *Anopheles* in all four communes in the rainy season, in the absence of agriculture. *Anopheles* needs to be identified to species level in each of the different communes, especially in the context of the invasion of *Anopheles stephensi* into eastern Africa [61]. The presence of *Anopheles* larvae in man-made containers suggests that *Anopheles* species can adapt to diverse containers, which in turn indicates a heightened transmission risk of malaria in urban Kinshasa.

## Conclusion

*Aedes* spp*.* seem to be well established in all of the four communes of Kinshasa surveyed here, and are especially abundant in the suburban area of Mont Ngafula. This study, which to our knowledge is the first of its kind to be carried out in Kinshasa, shows that *Aedes* control strategies here need to target outdoor containers, specifically those designed for water storage, in the dry season, and tires in the rainy season. Additional insights into the ecology of adult *Aedes* mosquitoes and their susceptibility to insecticides will support the design of a comprehensive *Aedes* control strategy for the prevention of further outbreaks of arboviral diseases in Kinshasa.

## Supplementary Information

**Additional file 1: Table S1.** Distribution of inspected containers inside/outside the houses, Kinshasa 2018.

**Additional file 2.** DNA based species validation (methods, results and Figure S1).

## Data Availability

The datasets used and/or analyzed during the current study are available from the corresponding author on reasonable request.

## Abbreviations

DRC: Democratic Republic of Congo
INRB: Institut National de Recherche Biomédicale
BI: Breteau index
CI: Container index
HI: House index
aOR: Adjusted odds ratio
