# Supplementary material for: High Aedes spp. larval indices in Kinshasa, Democratic Republic of Congo
Source: Parasit Vectors. 2021 Feb 1;14:92. doi: 10.1186/s13071-021-04588-7 (PMC7852359; doi:10.1186/s13071-021-04588-7)
Supplement: Supplementary file 1 — Additional file 1: Table S1. Distribution of inspected containers inside/outside the houses, Kinshasa 2018. [file 13071_2021_4588_MOESM1_ESM.docx]

**Additional File 1. Table 1. Distribution of inspected containers inside/outside the houses**

|  | total | |  | lingwala | |  | ndjili | |  | mont ngafula | |  | kalamu | |
| --- | --- | --- | --- | --- | --- | --- | --- | --- | --- | --- | --- | --- | --- | --- |
| nr containers inspected per categorie | Rainy season (% external) | Dry season (% external) |  | Rainy season (% external) | Dry season (% external) |  | Rainy season (% external) | Dry season (% external) |  | Rainy season (% external) | Dry season (% external) |  | Rainy season (% external) | Dry season (% external) |
| Water storage tanks | 786 (50.8) | 294 (70.1) |  | 90 (77.8) | 14 (100) |  | 412 (23.3) | 120 (54.2) |  | 201 (77.6) | 133 (78.2) |  | 83 (92.8) | 27 (85.2) |
|  |  |  |  |  |  |  |  |  |  |  |  |  |  |  |
| Small water deposits | 3266 (59.2) | 1107 (75.6) |  | 466 (75.5) | 107 (93.5) |  | 1835 (41.8) | 472 (64.0) |  | 685 (79.9) | 408 (78.9) |  | 280 (95.4) | 120 (94.2) |
|  |  |  |  |  |  |  |  |  |  |  |  |  |  |  |
| Rubbish/discards | 432 (98.4) | 101 (100) |  | 143 (99.3) | 35 (100) |  | 109 (95.4) | 13 (100) |  | 125 (99.2) | 37 (100) |  | 55 (100) | 16 (100) |
| Bamboo holes | 5 (100) | 0 |  | 0 | 0 |  | 1 (100) | 0 |  | 3 (100) | 0 |  | 1 (100) | 0 |
| Artificials not destroyable | 13 (100) | 5 (100) |  | 0 | 0 |  | 0 | 0 |  | 13 (100) | 5 (100) |  | 0 | 0 |
|  |  |  |  |  |  |  |  |  |  |  |  |  |  |  |
| Used tires | 565 (99.6) | 145 (99.3) |  | 120 (100) | 24 (100) |  | 189 (98.9) | 59 (98.3) |  | 133 (100) | 49 (100) |  | 123 (100) | 13  (100) |
| Ground pools | 12 (91.7) | 5 (80.0) |  | 2 (100) | 0 |  | 4 (100) | 1 (0.0) |  | 4 (100) | 2 (100) |  | 2 (50.0) | 2 (100) |
