# Supplementary material for: High Aedes spp. larval indices in Kinshasa, Democratic Republic of Congo
Source: Parasit Vectors. 2021 Feb 1;14:92. doi: 10.1186/s13071-021-04588-7 (PMC7852359; doi:10.1186/s13071-021-04588-7)
Supplement: Supplementary file 2 — Additional file 2. DNA based species validation (methods, results and Figure S1). [file 13071_2021_4588_MOESM2_ESM.docx]

**Additional File 2: DNA-based species validation**

In total, 42 and 36 specimens of *Ae. albopictus* and *Ae. aegypti*, respectively, were randomly selected to validate the morphological identifications. Sanger sequencing of the 658 bp COI standard barcode (Folmer et al. 1994; Hebert et al. 2003) was performed as described in the EPPO PM7/129(1) publication (EPPO 2016; LCO1490 and HCO2198 universal primers). Amplifications were carried out in a 20 µl reaction mixture containing 2 µl of DNA template, 2 µl of 10X buffer, 1.5 mM MgCl2, 0.2 mM dNTP, 0.4 µM of each primer, and 0.03 units/µl of Platinum^TM^ Taq DNA Polymerase (Invitrogen^TM^). PCR products and negative controls were checked on a 1.5% agarose gel, using a UV transilluminator and the MidoriGreenTM Direct (NIPPON Genetics Europe) method. Positive amplicons were purified using the ExoSAP-IT^TM^ protocol and sequenced in both directions on an ABI 3230xl capillary DNA sequencer using BigDye Terminator v3.1 chemistry (ThermoFisher Scientific).

Geneious^®^ R11 (Biomatters Ltd., Auckland, New Zealand) was used to check for the quality of the sequencing output, to assemble the paired bi-directional strands after correction, and to extract one consensus sequence per specimen. Subsequently, the COI consensus sequences were compared against the Identification System of BOLD, with Species Level Barcode Records option ([www.boldsystems.org](http://www.boldsystems.org)). Haplotypes and nucleotide diversities over loci were evaluated using Arlequin 3.5 (Excoffier and Lischer, 2010).

To perform further analyses, a list of *Aedes* species of the subgenus Stegomyia of medical importance found to occur in the Afrotropical region was established by consulting the WRBU and the APHC databases ([http://www.wrbu.org/aors/africom_Keys.html#](http://www.wrbu.org/aors/africom_Keys.html); <https://phc.amedd.army.mil/>). For each of these species, publicly available COI sequences from BOLD were downloaded, cleaned and aligned using ClustalW in Geneious® R11. The alignment was trimmed to only retain the standard barcode region. Afterwards, duplicate sequences (i.e. identical sequences according to Geneious) per species and per sampling country were removed, as well as barcodes of less than 400 bp. Next, the generated consensus sequences, as well as sequences of an outgroup (*Culex quinquefasciatus*), were added to the alignment. Based on Kimura 2-parameter (K2P) distances (Kimura 1980), a rooted Neighbour-Joining tree (NJ) was constructed using MEGA7 (Kumar et al. 2016; Saitou and Nei 1987), with branch support assessed by 500 bootstrap (BS) replicates (Felsenstein 1985). Clustering of the generated sequences in relation to the other species in the dataset, and the respective BS support of each cluster, was examined. For *Ae. albopictus* and *Ae. aegypti*, however, the amount of barcodes in the database was still substantial. For readability purposes, a second NJ-tree was constructed using a subselection of the barcodes. Finally, the average interspecific K2P distances, as well as the maximum observed K2P distances between conspecific sequences, were finally calculated among COI sequences with the package Spider (Brown et al. 2012) using R 3.6.2.

**Results of species identification validation:**

The morphological identifications were validated by comparing the generated sequences against the Identification System of BOLD, with Species Level Barcode Records. The obtained similarity percentages ranged from 99.69 to 100%. In total, five and 14 haplotypes were identified within the databases of generated COI barcodes of *Ae. albopictus* and *Ae. aegypti*, respectively. The average nucleotide diversities over loci were of 0.001 ± 0.001 and of 0.006 ± 0.004, for *Ae. albopictus* and *Ae. aegypti*, respectively. The generated sequences were deposited in GenBank with following accession numbers: MT345349-MT345426.

In the Afrotropical region, six *Aedes* species of the subgenus Stegomyia of medical importance are reported. After filtering, a total of 1,474 COI sequences were retained in the final analyses (Table S1; Median: 38; Mean: 246 sequences per species). Pairwise K2P distances between species varied from 7.29 to 11.59%, and the largest intraspecific K2P distance was 12.69% for *Ae. albopictus* (Table S1 – Fig. S1). The pairwise genetic distances between *Ae. aegypti* / *Ae. albopictus* and all the other species were the largest in the database (> 11%; Table S2 – Fig. S1). In the NJ-tree based on COI sequences, species formed well-supported clusters with high bootstrap support (BS; Figure 5 in main manuscript – full NJ-tree can be provided on request). The five and 14 haplotypes of *Ae. albopictus* and *Ae. aegypti*, respectively, are clustering only with conspecific sequences from specimen collected worldwide, supported with maximum BS support (Figure 5 in main manuscript).

Table S2. List of medically important *Aedes* mosquito species of the subgenus Stegomyia occurring in the Afrotropical region, following WRBU and APHC, including the overall maximum observed intraspecific Kimura two-parameter (K2P) distances among COI sequences. Sequences were extracted from BOLD (public). In **bold**: number of newly sequenced specimens from this study. n.a.: not applicable.

| Species | Number of COI sequences included in the analysis | Average inter-specific K2P distance (%) | Max observed K2P distance between conspecific sequences (%) |
| --- | --- | --- | --- |
| *Aedes aegypti* | 650 + **36** | 11.22 | 4.98 |
| *Aedes africanus* | 50 | 9.18 | 6.32 |
| *Aedes albopictus* | 649 + **42** | 11.59 | 12.69 |
| *Aedes bromeliae* | 25 | 8.03 | 2.77 |
| *Aedes luteocephalus* | 1 | 7.29 | n.a. |
| *Aedes vittatus* | 21 | 8.61 | 1.44 |

Figure S1. Boxplot of the inter- and intraspecific genetic distances (K2P) between species listed in Table S2 (1,474 COI sequences), indicating the existence of a barcode gap, with interspecific distance being larger than intraspecific distance. The median is indicated by the horizontal line and the range as the vertical dashed lines (upper and lower 1.5 IQR quartiles). Outliers are displayed as open circles (due to overlap, they look like bold vertical lines). Box represents the upper and lower quartiles.


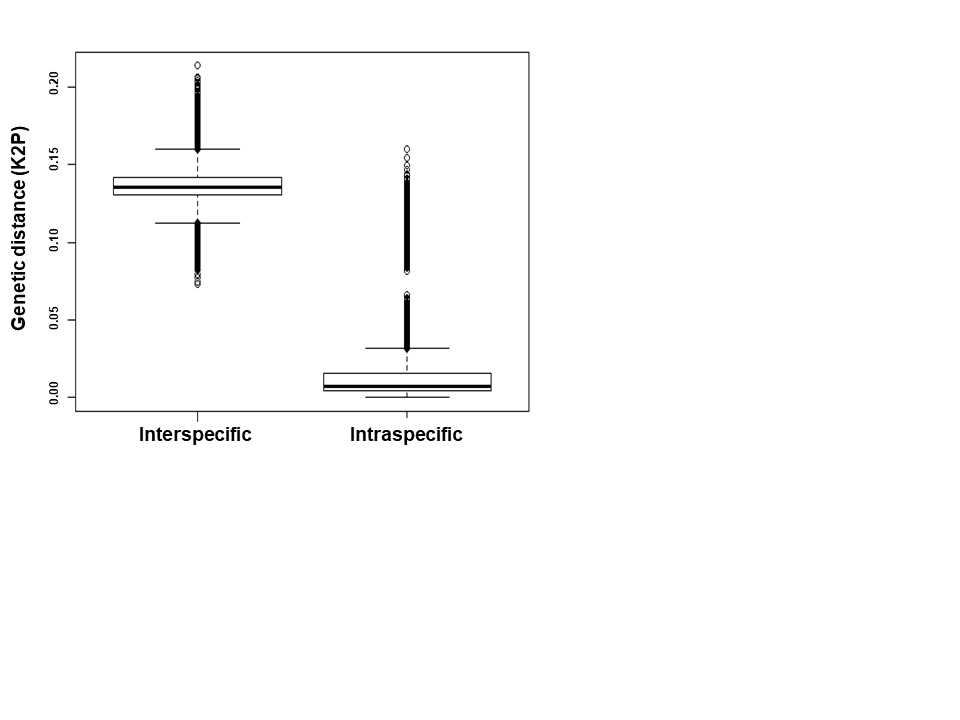


**References**

Folmer, O.; Black, M.; Hoeh, W.; Lutz, R.; Vrijenhoek, R. DNA primers for amplification of mitochondrial cytochrome c oxidase subunit I from diverse metazoan invertebrates. Mol Mar Biol Biotechnol 1994, 3, 294-299.

Hebert P.D.N., Cywinska A., Ball S.L. and deWaard J.R. (2003). Biological identifications through DNA barcodes. Proc. R. Soc. Lond. B. 270: 313–321. Available at https://doi.org/10.1098/rspb.2002.2218.

EPPO. PM 7/129 (1) DNA barcoding as an identification tool for a number of regulated pests. EPPO bulletin 2016, 46, 501-537, <https://doi.org/510.1111/epp.12344>.

Huang, Y. M. Adult identification key to the genus Aedes in the AFRICOM Afrotropical Region, with emphasis on medically important mosquitoes. http://www.wrbu.org/aors/africom_Keys.html#

Kimura, M. A simple method for estimating evolutionary rates of base substitutions through comparative studies of nucleotide sequences. Journal of Molecular Evolution 1980, 16, 111-120, doi:10.1007/BF01731581.

Excoffier, L. and H.E. L. Lischer (2010) Arlequin suite ver 3.5: A new series of programs to perform population genetics analyses under Linux and Windows. Molecular Ecology Resources. 10: 564-567.

Kumar, S.; Stecher, G.; Tamura, K. MEGA7: Molecular Evolutionary Genetics Analysis Version 7.0 for Bigger Datasets. Mol Biol Evol 2016, 33, 1870-1874, doi:10.1093/molbev/msw054.

Saitou, N.; Nei, M. The neighbor-joining method: a new method for reconstructing phylogenetic trees. Mol Biol Evol 1987, 4, 406-425, doi:10.1093/oxfordjournals.molbev.a040454.

Felsenstein, J. Confidence Limits on Phylogenies: An Approach Using the Bootstrap. Evolution 1985, 39, 783-791, doi:10.1111/j.1558-5646.1985.tb00420.x.

Brown, S.D.; Collins, R.A.; Boyer, S.; Lefort, M.C.; Malumbres-Olarte, J.; Vink, C.J.; Cruickshank, R.H. Spider: an R package for the analysis of species identity and evolution, with particular reference to DNA barcoding. Mol Ecol Resour 2012, 12, 562-565, doi:10.1111/j.1755-0998.2011.03108.x.
